# Positive *Helicobacter pylori* status is associated with better overall survival for gastric cancer patients: evidence from case-cohort studies

**DOI:** 10.18632/oncotarget.18758

**Published:** 2017-06-28

**Authors:** Xuqian Fang, Kun Liu, Jialin Cai, Fangxiu Luo, Fei Yuan, Peizhan Chen

**Affiliations:** ^1^ Translational Medicine Research Center, Ruijin Hospital North, Shanghai Jiao Tong University School of Medicine, Shanghai, P. R. China; ^2^ Department of Pathology, Ruijin Hospital North, Shanghai Jiao Tong University School of Medicine, Shanghai, P. R. China; ^3^ Department of Surgery, Ruijin Hospital North, Shanghai Jiao Tong University School of Medicine, Shanghai, P. R. China

**Keywords:** Helicobacter pylori, gastric cancer, overall survival, disease-free survival, meta-analysis

## Abstract

*Helicobacter pylori* (*H. pylori*) infection increases the gastric cancer risk; however, the influences of *H. pylori* infection status on the outcomes for gastric cancer patients have not yet clearly defined. Herein, we systematically assessed the epidemiological studies regarding the associations between the *H.pylori* infection status at diagnosis and the prognosis for gastric cancer patients with the meta-analysis methods. Thirty-three eligibility studies with 8,199 participants that had determined the *H.pylori* infection status and the outcomes for gastric cancer patients were identified through searching the PubMed and MEDLINE databases updated to March 1^st^, 2017. The random-effects model suggested that positive H. pylori infection was associated with better overall survival with the pooled hazard ratio (HR) was 0.79 [95% confidence interval (CI) = 0.66-0.93; Q = 134.86, df = 32, P-heterogeneity < 0.001; *I*^2^ = 76.3%] compared to negative patients. The association was found to be more prominent in studies with higher quality, longer following-up time and more sensitive detection methods. An inverse but not statistically significant association between the H.pylori status and the disease-free survival of the patients (pooled HR = 0.84, 95% CI = 0.61-1.05;Q = 30.48, df = 11, P-heterogeneity = 0.001; *I*^2^ = 63.9%) was found, while no significant association was noticed in any subgroup analyses. These results suggested that gastric cancer patients with positive *H.pylori* infection status at diagnosis have better overall survival compared to negative; however, more studies are warranted to confirm the results and elucidate the underlying mechanisms.

## INTRODUCTION

In spite of a declining incidence rate, gastric cancer remains a major public health issue as it ranks as the third most common cause of cancer deaths worldwide [1]. It was estimated that gastric cancer leads to about 723,100 deaths in 2012 worldwide with more than half of the cases occurred in the Asians [1, 2]. For gastric cancer patients, surgical resection followed with chemotherapy are still the mainly effective therapy methods, especially for those at earlier stages [3]. It has been reported that clinical characteristics, including age, tumor location, invasion depth, lymph node involvement, and distant metastasis status are important prognosis factors for gastric cancer patients; however, these factors only account for a small proportion of the prognosis heterogeneity among the patients [4]. More prognostic factors are warranted to guide the clinical treatments and identify those patients who need more intensive treatments and following-up to improve the outcomes for gastric cancer patients.

*H. pylori,* a bacteria colonizing in the stomach, is a well-known carcinogen for gastric cancer and *H.pylori* infection leads to an 7-10 fold increased gastric cancer risk [5]. It was estimated that about 89% of the new noncardia gastric cancer cases were caused by the bacteria worldwide [6]. In healthy, asymptomatic individuals, *H. pylori* eradication treatment could significantly decrease gastric cancer risk [7]. As a risk factor closely related to gastric cancer development, influences of *H. pylori* infection status on the prognosis of gastric cancer patients have been investigated. Many epidemiological studies have reported that gastric cancer patients with positive *H. pylori* infection status showed a better overall survival (OS) compared to those without infection [8-10]; however, conflicting results were found for other studies that reported no significant association between the *H. pylori* infection status at diagnosis and the outcomes for gastric cancer patients [11-13]. As there the influences of *H.pylori* infection on the clinical outcomes of gastric cancer patients have not yet well established, we aimed to systematically evaluate the associations between the *H.pylori* infection status at diagnosis and the prognosis of the gastric cancer patients through meta-analysis of the published epidemiological studies in the current study.

## RESULTS

### Eligibility studies identification

**Figure 1:**
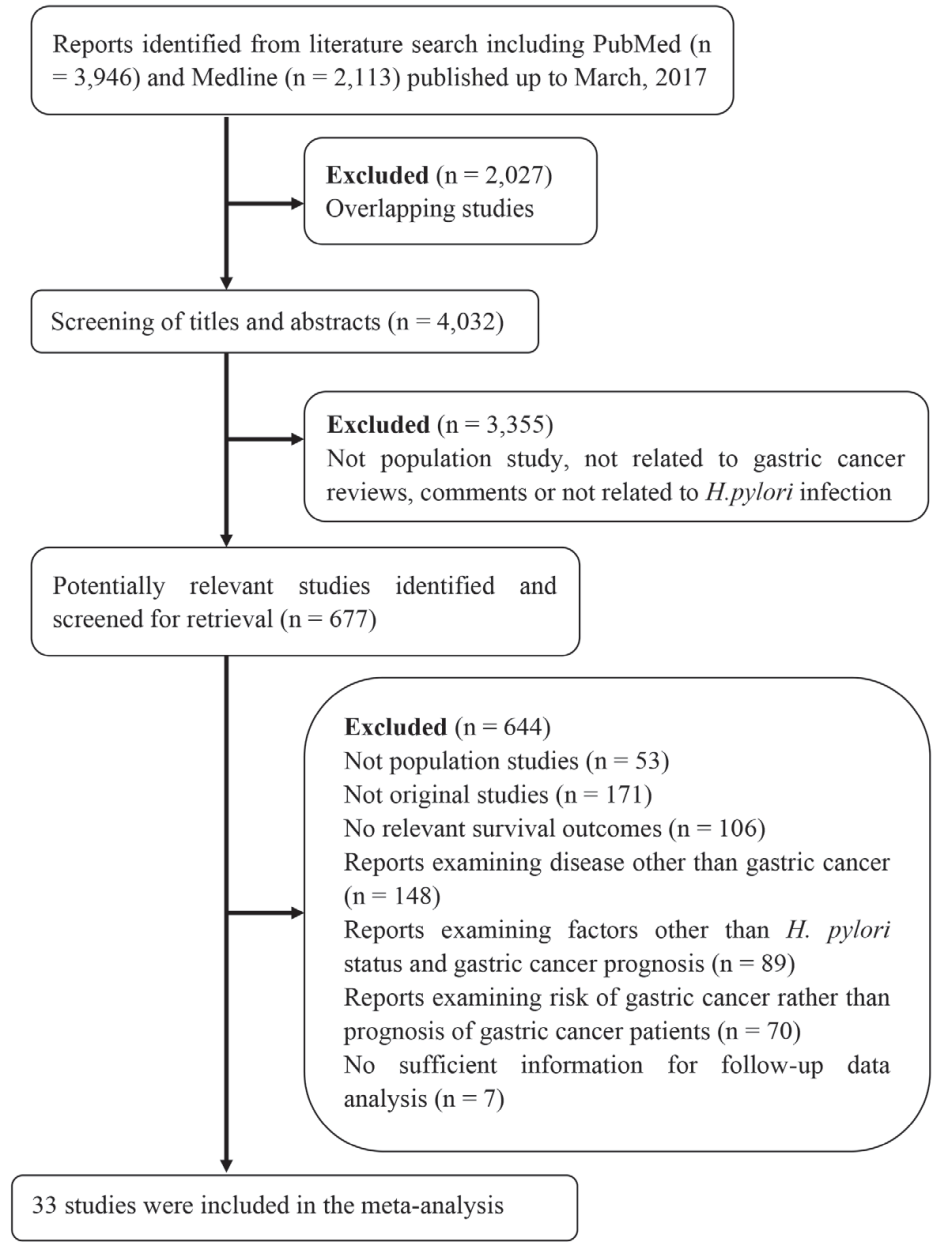
Working flow chart summarizing the studies identification and selection procedure for the meta-analysis studies

The working flow chart for the eligibility studies identification is presented as Figure 1. From the electronic databases search, a total of 4,032 unique studies that published updated to March 1^st^, 2017 were identified with the predefined terms. Through checking the title and abstracts, 3,355 clearly irrelevant studies were excluded. 677 potential studies were further checked with the abstract or the full-length of reports and 644 studies that did not fully meet the inclusion criteria were excluded from the meta-analysis. 33 studies with a total of 8,199 patients that fully met the inclusion criteria were included in the current meta-analysis studies [8-40].

### The study characteristics and quality assessment

Detailed information of the eligibility studies was provided as Table 1. All the 33 identified studies have determined the associations between the *H. pylori* status at diagnosis and the OS, while only 12 studies determined the disease-free survival (DFS) of gastric cancer patients (Table 1). For the included studies, 26 were performed in Asian countries [10-16, 18-26, 28, 29, 32, 34-40], and 7 in non-Asian countries [8, 9, 17, 27, 30, 31, 33]. The sample size of the studies was ranged from 61 to 794 patients, with the *H.pylori* positive rate of the patients was ranged from 17.5% to 95.5%. The median following-up for the patients was ranged from 10.4 to 144 months for the included studies. Based on the Newcastle-Ottawa quality assessment scale, the quality scores for the eligibility studies were ranged from 6 to 10. Seventeen studies with quality score > 7 were classified as high-quality studies, while sixteen studies with quality score ≤ 7 were classified as low-quality studies (Supplementary Table 1).

**Table 1:** Characteristics of the 33 included studies that have determined the associations between *H. pylori* status (positive *vs.* negative) and the prognosis for gastric cancer patients.

Source	Clinical stage, No. of patients	Study country	Median following-up time (range)	*H. pylori* detection method	*H. pylori* Positive (%)	HR (95% CI) for *H. pylor*i positive *vs*. negative	Quality score
Lee *et al*, 1995	All stages, 128	China	36 months	Serologic analysis	82 (64.10%)	OS: 0.58 (0.35-0.94)^*,u^	7/10
Kurtenkov *et al*, 2003	Early stage (I and II), 87	Estonia	NA	Serologic analysis	58 (66.67%)	OS: 0.37 (0.20-0.67)^*, u^	6/10
Meimarakis *et al*, 2006	All stages, 166	Germany	53.0 (1-146) months	Serologic analysis, histological examination, Bacterial culture	125 (75.30%)	OS: 0.50 (0.31-0.82)^m^ DFS: 0.46 (0.29-0.75)^m^	10/10
Marrelli *et al*, 2009	All stages, 297	Italy	62 (1-220) months	Serologic analysis, PCR	256 (86.20%)	OS: 0.40 (0.23-0.71)^m^	10/10
Qiu *et al*, 2010	All stages, 157	China	24.4 (0.2-81.8) months	PCR	82 (52.23%)	OS: 1.09 (0.70-1.68)^*,u^ DFS: 1.13 (0.67-1.92)^*,u^	6/10
Gan *et al*, 2011	All stages, 794	China	50 months	Histological examination	239 (30.10%)	OS: 0.87 (0.70-1.08)^m^	9/10
Santos *et al*, 2011	All stage, 68	Brazil	65.6 (6.7-207.3) months	Histological examination	34 (50%)	OS: 0.68 (0.40-1.16)^*,u^	7/10
Syrios *et al*, 2012	Stage IV, 218	Greece	NA	Serologic analysis	76 (34.86%)	OS: 0.83 (0.56-1.23)^u^	6/10
Kang *et al*, 2012	All stage, 274	Korea	144 (120-184) months	Histological examination	166 (60.58%)	OS: 0.29 (0.20-0.41)^m^	9/10
Chen *et al* , 2012	All stages, 120	China	NA	PCR	21 (17.50%)	OS: 1.54 (0.73-3.24) ^u^	8/10
Chio *et al*, 2012	Advanced or metastatic, 61	Korea	NA	Histological examination	18 (29.51%)	OS: 0.65 (0.34-1.23)^*,u^	6/10
Hur *et al*, 2012	All stage, 174	Korea	NA	Histological examination, serologic analysis	111 (63.79%)	OS: 0.99 (0.45-2.17)^*,u^ DFS: 0.57 (0.28-1.05)^*,u^	7/10
Wang , *et al* 2013	All stages,261	China	Range, 35-59 months	Histological examination	188 (72.03%)	OS: 0.49 (0.27–0.89)^m^ DFS: 0.56 (0.31-1.00)^m^	9/10
Li *et al*, 2013	All stages, 162	China	35.3 (1.7-71.9) months	Histological examination	75 (46.29%)	OS: 1.71 (1.11-2.66)^m^ DFS: 1.68 (1.05-2.69)^m^	8/10
Posteraro *et al*, 2013	All stages, 110	Italy	52.9 (1-158) months	PCR	86 (78.18%)	OS: 1.15 (0.57-2.30)^u^ DFS: 0.89 (0.47-1.69)^u^	8/10
Gong *et al*, 2014	All stages, 308	Korea	70.7±41.5 months	Serologic analysis	259 (84.09%)	OS: 1.06 (0.50-2.24)^u^	8/10
Fang *et al*, 2014	With malignant ascites, 347	China	10.4 (0.3-60.1) months	NA	213 (81.30%)	OS: 1.27 (1.06-1.52)^m^	7/10
Roberts *et al*, 2014	All stages, 79	West Indies	NA	Histological examination	15 (19.48%)	OS: 0.46 (0.14-1.51)^u^	7/10
Ling *et al*, 2014	All stages, 300	China	28 (11-59) months	NA	165 (55.00%)	OS: 0.46 (0.11-1.93)^m^ DFS: 0.57 (0.34-0.96)^m^	7/10
Lian *et al*, 2014	All stages, 101	China	30.7 (2.7-60) months	Serologic analysis	64 (63.40%)	OS: 0.49 (0.27-0.86)^m^ DFS: 0.41 (0.24-0.85)^m^	8/10
Kim *et al*, 2014	All stages, 533	Korea	NA	Serologic analysis, histological examination	509 (95.50%)	OS: 0.51 (0.07-3.70)^m^ DFS: 0.78 (0.19-3.21)^m^	9/10
Shen *et al,* 2015	All stages, 136	China	NA	NA	126 (92.65%)	OS: 0.70 (0.39-1.24)^u^	6/10
Garcia-Gonzalez et al, 2015	All stages, 558	Spain	12.5 (1.3-124) months	Urease test, histological examination, serologic analysis	381 (68.28%)	OS: 1.03 (0.84-1.25)^u^	8/10
Piao et al, 2015	All stages, 205	China	NA	Serologic analysis	117 (57.07%)	OS: 0.92 (0.57-1.50)^u^	7/10
Wei et al, 2015	All stages, 166	China	NA	NA	122 (73.49%)	OS: 1.14 (0.66-1.98)^u^	6/10
Wang et al, 2015	All stage, 82	China	NA	Urease test, histological examination, serologic analysis	44 (53.66%)	OS: 0.73 (0.12-1.35)^u^	8/10
Zhang et al, 2015	Early stage, 65	China	509 (201-1208) days	Serologic analysis	40 (61.53%)	OS: 1.17 (0.48-2.85)^m^ DFS: 0.91 (0.38-2.20)^m^	8/10
Zhao et al, 2016	All stages, 600	China	NA	NA	475 (79.17%)	OS: 2.52 (1.58-4.01)^u^	6/10
Zhou et al, 2016	All stage, 152	China	38.1 months	Urease test	70 (46.05%)	OS: 0.46 (0.233-0.909)^u^	8/10
Chen et al, 2016	All stage, 67	China	NA	NA	44 (65.67%)	OS: 0.39 (0.08-2.00)^u^	6/10
Postlewait et al, 2016	All stage, 559	China	49.8 months	NA	455 (81.40%)	OS: 0.54 (0.30-0.99)^m^	8/10
Liu et al, 2016	All stage, 297	China	Range: 0-95 months	NA	208 (70.00%)	OS: 1.26 (0.74-2.14)^m^ DFS: 1.37 (0.84-2.23)^m^	7/10
Tsai et al, 2017	All stage, 567	China	NA	Histological examination, serologic analysis	435 (76.72%)	OS: 0.75 (0.58-0.96)^m^ DFS: 0.97 (0.67-1.40)^*,u^	9/10

Abbreviations: 95% CI, 95% confidence interval; DFS, disease-free survival; *H. pylori*, *Helicobacter pylori*; HR, hazard ratio; NA, not available; OS, overall survival; u, univariate analysis result; m, multivariate analysis result.

*The estimate and its 95% CI were recalculated based on the provided information in the study.

### *H. pylori* status and overall survival of gastric cancer patients

**Figure 2:**
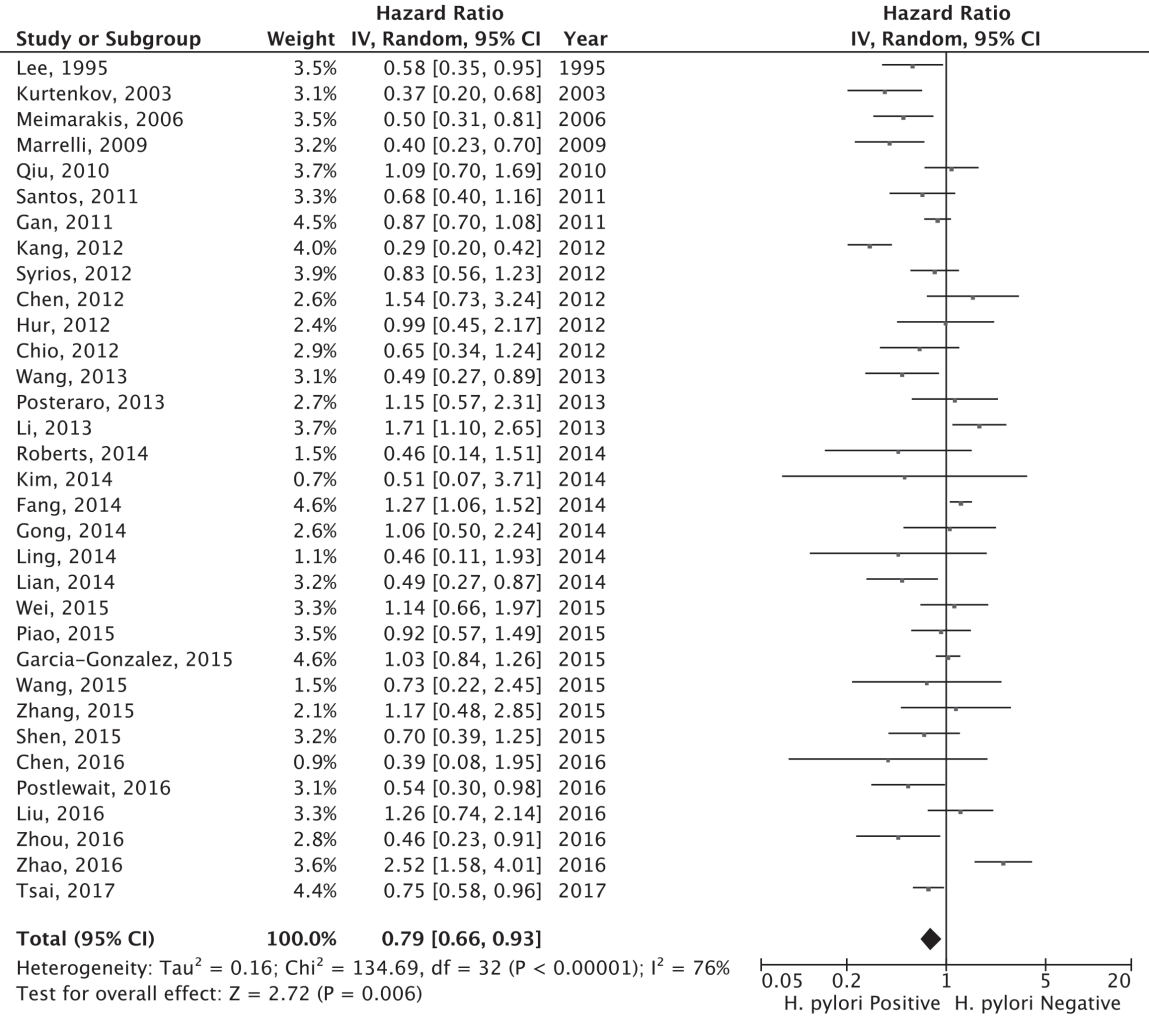
Forest plot for the association between *H. pylori* status (positive *vs.* negative) at diagnosis and overall survival of gastric cancer patients

**Figure 3:**
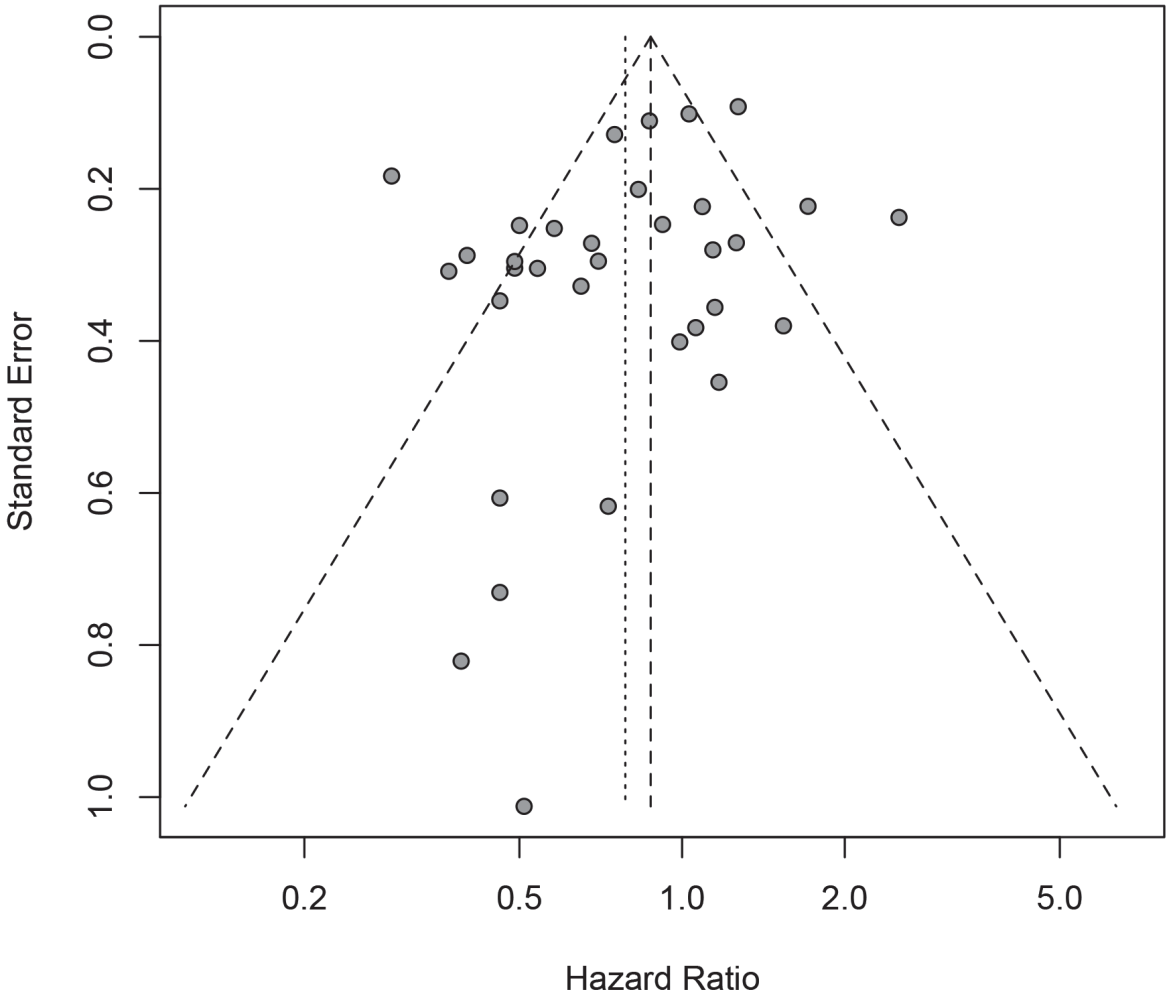
Funnel plot for the estimated risk between *H.pylori* infection status (positive *vs*. negative) at diagnosis and the overall survival of gastric cancer patients

Under the random-effects model, the pooled hazard ratio (HR) was 0.79 [95% confidential interval (95% CI) = 0.66-0.93; positive *vs*. negative; Figure 2] of the 33 eligibility studies that determined the influences of *H.pylori* infection status and OS of gastric cancer patients, suggesting that gastric cancer patients with positive *H. pylori* status was associated with better OS in relative to negative. Significant heterogeneity between the studies was found (Q = 134.86, df = 32, P-heterogeneity < 0.001; *I*^2^ = 76.3%). The sensitivity studies showed that no individual study significantly affected the overall estimate of the meta-analysis. We applied the Baujat plot to identify those studies that largely contributed to the heterogeneity between the studies, and found that three studies performed by Kang et al. [20], Fang et al. [11], and Zhao et al. [38] caused significant heterogeneity between the studies (Supplementary Figure 1). When these three studies were excluded, we still found that gastric cancer patients with positive *H.pylori* status at diagnosis were associated with better OS compared to those of negative status (pooled HR = 0.78, 95% CI = 0.67-0.90). A significant reduction of the heterogeneity between the studies was noticed for the 30 included studies (Q = 60.52, df = 29, P-heterogeneity = 0.005; *I*^2^ = 52.1%). No significant publication bias was noticed as suggested by the funnel plot (Egger’s test, *P* = 0.086 and Begg’s test, *P* = 0.337; Figure 3). The cumulative meta-analysis suggested that the pooled estimate was chronologically stable (Supplementary Figure 2).

To explore the potential heterogeneity between the studies, the subgroup and the meta-regression analyses were performed to identify the study level characteristics that might significant influence the pooled estimate. In the subgroup study, we found the association was more prominent in patients from non-Asian countries (pooled HR = 0.71, 95% CI = 0.52-0.97) in relative to those from Asian countries (pooled HR = 0.81, 95% CI = 0.65-1.00; Table 2). Studies with higher quality (pooled HR = 0.71, 95% CI = 0.52-0.97), longer follow-up time (pooled HR = 0.64, 95% CI = 0.47-0.87) and relative smaller sample size (pooled HR = 0.77, 95% CI = 0.61-0.97) showed more prominent associations between *H. pylori* status and the OS of gastric cancer patients compared to studies of lower quality, shorter follow-up time or relatively larger sample size (Table 2). In the stratification analyses of the *H. pylori* detection method, a significant association was found for those studies conducted with two or more *H. pylori* detection methods (pooled HR = 0.68, 95% CI = 0.48-0.95) or with serologic analysis (pooled HR = 0.70, 95% CI = 0.52-0.93); however, no significant association was found in those studies that only performed the histological examination or PCR detection tests (Table 2). The prognosis of gastric cancer patients might be influenced by the clinical stage. The pooled estimate from 14 studies with multivariate adjustment for the cofounders (including age, sex, tumor location, disease stage, and adjuvant treatment etc.) suggested that positive *H. pylori* was associated with improved OS of gastric cancer patients (pooled HR = 0.70, 95% CI = 0.52-0.94), whereas pooled estimate for 23 studies of univariate analysis showed a negative but non-statistically significant association between the *H.pylori* infection status and OS (pooled HR = 0.86, 95% CI = 0.71-1.04; Table 2). However, the meta-regression studies suggested the pooled estimates between the groups were not significantly different (data not shown). For all the stratified studies, significant heterogeneity between the studies was noticed and no significant publication bias was evident as suggested by the funnel plots in together with the Egger’s test or Begg’s test (Table 2). The sensitivity analyses suggested that none individual study significantly influenced the pooled estimate in any subgroup studies.

**Table 2:** Stratification analyses for the association between *H. pylori* infection (positive *vs*. negative) status at diagnosis and the overall survival of gastric cancer patients.

Stratification factor	No. of Studies/Patients		Random-effects model HR (95%CI)*	Q/df	P-heterogeneity	I^2^	Egger's test	Begg's test
Overall survival	33/8,199		0.79 (0.66-0.93)	134.86/32	< 0.001	76.3%	0.086	0.337
Disease stage								
Early stage	2/152		0.63 (0.20-1.93)	4.39/1	0.036	77.2%	NA	NA
Advanced stage	3/317		0.76 (0.55-1.05)	0.90/2	0.638	0.0%	0.191	0.117
All stage	29/7,768		0.80 (0.66-0.97)	125.56/28	< 0.001	77.7%	0.126	0.294
Region								
Asia	26/6,703		0.81 (0.65-1.00)	117.04/25	< 0.001	78.6%	0.235	0.343
Non-Asia	7/1,496		0.71 (0.52-0.97)	17.45/6	0.008	65.6%	0.099	0.293
Statistical methodology								
Multivariate analysis	14/4,723		0.70 (0.52-0.94)	88.27/13	< 0.001	85.3%	0.157	0.870
Univariate analysis	23/4,658		0.86 (0.71-1.04)	59.13/22	< 0.001	62.8%	0.265	0.476
Study quality								
Higher (Quality score > 7)	17/5,109		0.71 (0.56-0.91)	77.35/16	< 0.001	78.2%	0.414	0.680
Lower (Quality score ≤ 7)	16/3,090		0.88 (0.69-1.11)	48.10/15	< 0.001	68.8%	0.028	0.150
Median following-up time								
> 36 months	12/3,279		0.64 (0.47-0.87)	55.56/11	< 0.001	80.2%	0.577	0.784
≤ 36 months	21/4,920		0.92 (0.76-1.11)	54.65/20	< 0.001	63.4%	0.122	0.205
Sample size								
> 200	15/6,118		0.80 (0.62-1.04)	92.7/14	< 0.001	84.9%	0.261	0.299
≤ 200	18/2,081		0.77 (0.61-0.97)	39.41/17	0.002	56.9%	0.444	0.791
*H. pylori* detection method								
Serologic analysis		7/1,112	0.70 (0.52-0.93)	10.69/6	0.099	43.9%	0.941	0.881
Histological examination		8/1,873	0.69 (0.44-1.07)	45.55/7	< 0.001	84.6%	0.742	0.621
PCR		3/387	1.18 (0.85-1.65)	0.62/2	0.733	0.0%	0.433	0.117
Two or more methods		6/2,203	0.68 (0.48-0.95)	16.00/5	0.007	68.7%	0.183	0.573
NA or other		9/2,624	0.94 (0.65-1.35)	31.42/8	< 0.001	74.5%	0.148	0.022
*H.pylori* positive percent in gastric cancer patients								
> 64.0%		17/5,187	0.80 (0.63-1.01)	72.41	< 0.001	77.9%	0.081	0.249
≤ 64.0%		16/3,012	0.77 (0.59-1.00)	56.33/15	< 0.001	73.4%	0.860	0.418

Abbreviations: 95% CI, 95% confidence interval; HR, hazard ratio; *H. pylori*, *Helicobacter pylori*; NA, not available; PCR, Polymerase chain reaction.

*HR = 1 for negative *H. pylori* status;

### *H. pylori* status and disease-free survival of gastric cancer patients

**Figure 4:**
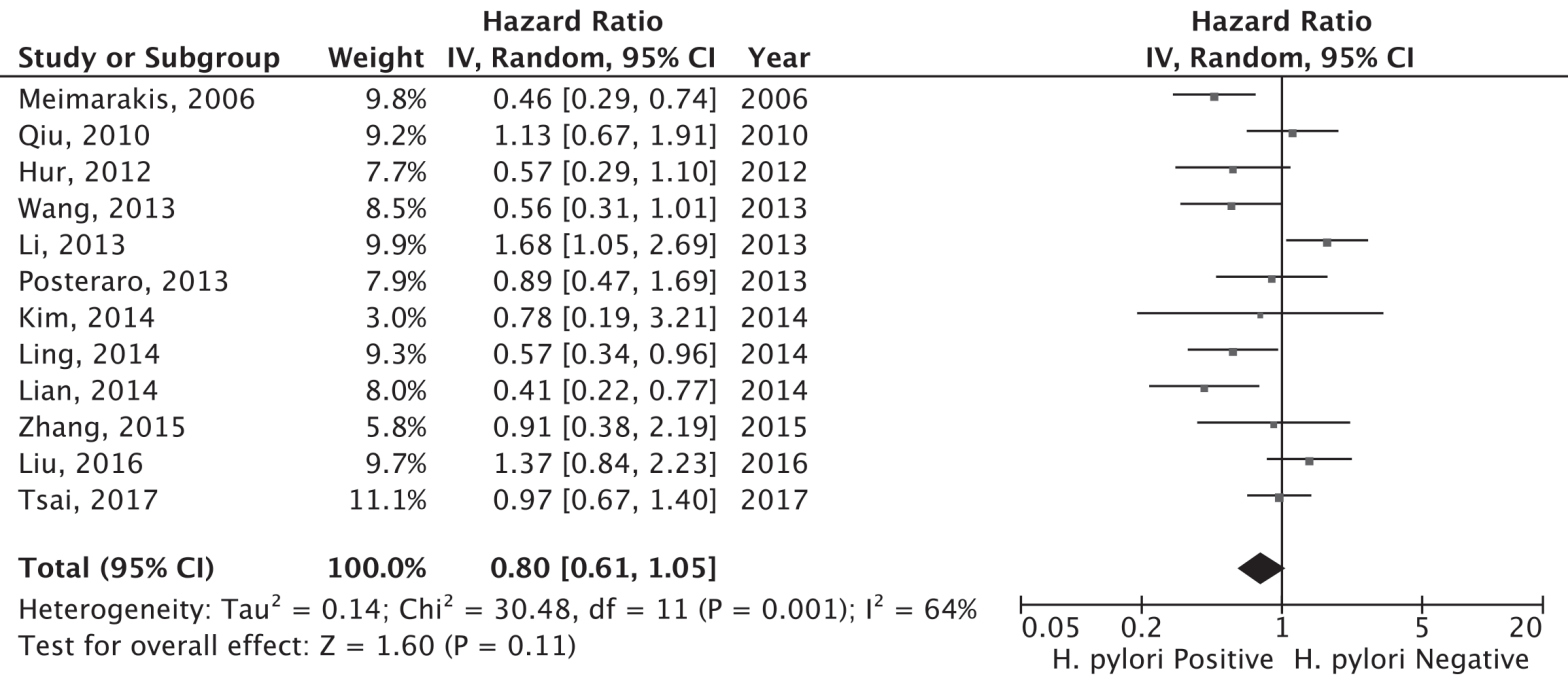
Forest plot for *H. pylori* status (positive *vs*. negative) and disease-free survival for gastric cancer patients

**Figure 5:**
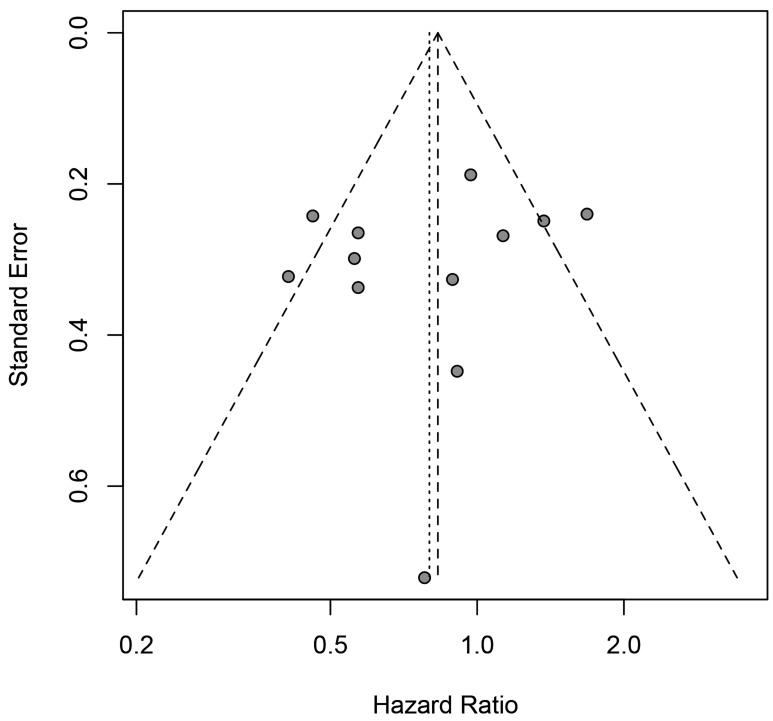
Funnel plot for the estimated risk between *H.pylori* infection status (positive *vs*. negative) at diagnosis and the disease-free survival of gastric cancer patients

Twelve studies with a total of 2,893 gastric cancer patients have determined the associations between the *H. pylori* status and the DFS for gastric cancer patients. The pooled estimate suggested that patients with positive *H. pylori* status showed an improved but not statistically significant better DFS (pooled HR = 0.80, 95% CI = 0.61-1.05; Figure 4) compared to those without *H. pylori* infection. Significant heterogeneity between the studies was evident (Q = 30.48, df = 11, *P* = 0.001, *I*^2^ = 63.9%). None of the individual studies significantly affect the pooled estimate as suggested by the sensitivity studies. With the Baujat plot, we noticed that four studies performed by Meimarakis et al. [9], Li et al. [12], Lian et al. [24] and Liu et al. [25] largely contributed to the overall heterogeneity between the studies (Supplementary Figure 3). The pooled estimate for the remained eight studies was 0.80 (95% CI = 0.65-0.98) and no significant heterogeneity between the studies was noticed (Q = 6.97, df = 7, *P* = 0.432; *I*^2^ = 0.0%). The funnel plot suggested that no significant publication was noticed for the included studies (Figure 5; Egger’s test, *P* = 0.454 and Begg’s test, *P* = 0.493). The cumulative meta-analysis suggested that the pooled estimate was chronically stable for the association between the *H.pylori* status and the DFS of gastric cancer patients (Supplementary Figure 4).

In the stratification studies, we found no significant association between the *H.pylori* infection status and the DFS for gastric cancer patients in any subgroup study stratified by the study region, *H.pylori* infection method, sample size, *H.pylori* positive percent, study quality, following-up time or whether the estimate was adjusted by covariates (Table 3). The meta-regression also suggested that none of these study-level characteristics significantly influenced the pooled estimate (data not shown). For all the subgroup studies, no significant publication bias was identified and the sensitivity studies suggested that none of the individual study significantly affected the pooled estimates (Table 3).

**Table 3:** Stratification analyses for the association between the *H. pylori* infection (positive vs. negative) status at diagnosis and the disease-free of survival of gastric cancer patients.

Stratification factor	No. of Studies/Patients	Random-effects model HR (95%CI)*	Q/df	*P*-heterogeneity	*I*^2^	Egger's test	Begg's test
Overall	12/2,893	0.80 (0.61-1.05)	30.48/11	0.001	63.9%	0.454	0.493
Region							
Asia	10/2617	0.84 (0.63-1.14)	23.79/9	0.005	62.2%	0.327	0.245
Non-Asia	2/276	0.62 (0.33-1.17)	2.63/1	0.105	62.0%	NA	NA
Statistical methodology							
Univariate analysis	7/1,631	0.96 (0.72-1.28)	12.40/6	0.054	51.6%	0.388	0.652
Multivariate analysis	8/1,885	0.75 (0.49-1.14)	26.81/7	0.004	73.9%	0.720	0.805
Study quality							
Higher (Quality score > 7)	8/1,965	0.76 (0.53-1.11)	21.73/7	0.003	67.8%	0.624	0.621
Lower (Quality score ≤ 7)	4/928	0.86 (0.55-1.36)	8.37/3	0.039	64.1%	0.391	0.497
Median following-up time							
> 36 months	4/699	0.79 (0.42-1.48)	16.30/3	0.001	81.6%	0.825	1.000
≤ 36 months	8/2,194	0.81 (0.60-1.09)	14.11/7	0.049	50.4%	0.450	0.458
*H. pylori* detection method							
PCR	2/267	1.03 (0.68-1.54)	0.32/1	0.572	0.0%	NA	NA
Histological examination	3/597	0.83 (0.38-1.80)	11.06/2	0.004	81.9%	0.233	0.602
Serologic analysis	2/166	0.57 (0.27-1.24)	2.09/1	0.149	52.1%	NA	NA
NA	2/597	0.89 (0.38-2.10)	5.82/1	0.016	82.8%	NA	NA
Two or more	3/1,266	0.69 (0.38-1.25)	5.92/2	0.052	66.2%	0.864	0.602
Sample size							
> 200	5/1,958	0.83 (0.58-1.19)	8.40/4	0.078	52.4%	0.638	0.624
≤ 200	7/935	0.78 (0.51-1.19)	21.91/6	0.001	72.6%	0.642	0.652
H.pylori positive percent in gastric cancer patients							
> 64.0%	6/1,934	0.80 (0.55-1.14)	12.43/5	0.029	59.8%	0.774	0.851
≤ 64.0%	6/959	0.80 (0.50-1.26)	18.00/5	0.003	72.2%	0.375	0.573

Abbreviations: 95% CI, 95% confidence interval; *H. pylori*, *Helicobacter pylori*; HR, hazard ratio; NA, not applicable; PCR, polymerase chain reaction.

*HR = 1 for negative *H. pylori* status.

## DISCUSSION

It has been more than 30 years since the discovery of *H. pylori* infections in the stomach of human [41]. The International Agency for Research on Cancer (IARC) has classified *H. pylori* as a type 1 carcinogen for gastric cancer [42]. *H. pylori* infection leads to the chronic gastritis, peptic ulcers of the stomach, and a chronic inflammatory process that may increase the risk of metaplastic epithelium and subsequent gastric cancer [43]. A previous published meta-analysis for randomized controlled trials confirmed that, *H. pylori* eradication reduced the risk for gastric cancer in those healthy and asymptomatic individuals; however, for those with preneoplastic conditions, *H. pylori* eradication have limited effects on the reduction of gastric cancer risk [7]. Thus, *H. pylori* infection is a risk factor for development of gastric cancer, and *H.pylori* eradication has preventive effects in the gastric cancer development. In the current study, through systematically evaluating the epidemiological studies, we reported that gastric cancer patients with positive *H. pylori* showed better OS compared to those without the infection at the time of diagnosis. As the *H.pylori* infection could be easily detected before the resection treatment, it could be served as an independent prognostic prediction biomarker for OS in gastric cancer patients. Moreover, the detection of *H.pylori* status could have substantial effects on disease outcomes as the results suggested that gastric cancer patients with negative *H. pylori* infection at diagnosis might need more stringent treatments.

Owing to the long evolutionary cohabitation of *H. pylori* with humankind, it has been suggested that this bacterium might confer some benefits to individuals. For example, *H. pylori* is a protective factor for symptom severity, symptom evolution and treatment response in gastroesophageal reflux disease [44]. *H. pylori* infection also protect infected individuals from development of atopic diseases through the modulation of the activities for Treg cells [45]. For gastric cancer, our current study suggested that gastric cancer patients with positive *H.pylori* have favorable outcomes through integrating the epidemiological studies. This seems paradoxical but still have biological underlying mechanisms. First, the immunological consequences of *H. pylori* infected might contribute to the favorable outcomes for gastric cancer patients. *H.pylori* infection-induced mucosal inflammation is Th1 mediated [46], and tumor infiltration by Th1 cells is associated with improved prognosis of patients [47]. *H.pylori*-infected macrophages also induce Th17 cell differentiation and increased Th17 cells have been reported to predict better survival in gastric cancer patients [48]. Persistent *H. pylori* infection might also exert protective effects by the secretion of *H. pylori* neutrophil-activating protein (HP-NAP). HP-NAP is a major virulence factor and a powerful inducer for inflammatory reaction and Th1-polarized immune response [49, 50]. Recent studies indicated that HP-NAP inhibits the growth of bladder cancer through activating the cytotoxic Th1 responses [51]. Moreover, expression of secreted HP-NAP by adenovirus has been shown to enhance the antitumor activity of these viruses in the treatment of metastatic breast cancer and neuroendocrine tumors, respectively [52, 53]. Second, several studies have suggested that *H.pylori* infection might increase the microsatellite instability (MSI) [54, 55], which was reported to be associated with better outcomes for gastric cancer patients [56]. Third, the signal regulated by the *H.pylori* in the gastric cells might contribute to the outcomes for gastric cancer patients, Zhou et al. reported that *H. pylori* reduced the microRNA-141 expression, which increased the expression of its target gene KEAP1 and thus enhanced the sensitivity of the gastric cancer cells to cisplatin [57]. Choi et al. reported that *H.pylori* positive patients with advanced or metastatic gastric cancer had a better response to 5-FU and cisplatin adjunct chemotherapy and an improved overall prognosis compared with patients without *H.pylori* infection [16]. At last, clinical studies indicated that patients with negative *H.pylori* infection was correlated with the advanced stage of gastric cancer patients [58]. Moreover, several studies also suggested that gastric cancer with negative *H.pylori* infection status were usually found in the proximal (cardia or fundus) compared to the distal (antrum or corpus) for *H.pylori* positive status [9, 28, 29], and it has been reported that proximal location was correlated with poorer OS for gastric cancer patients compared to distal location [59, 60]. All these might contribute to the improved OS of *H. pylori* positive gastric cancer patients; however, more studies are warranted to fully elucidate the protective roles of the *H.pylori* in the progression of gastric cancer patients and the underlying mechanisms.

Compared to a previous meta-analysis study performed by Wang et al. [61], the current study provided stronger evidence for the association between *H.pylori* infection status at diagnosis and the OS for gastric cancer patients with larger sample size. In the subgroup analyses, we found the association magnitude between the *H.pylori* infection status and the OS was different between the Asian and non-Asian populations (Table 2). We proposed that the baseline characteristics of the populations including genetic, dietary habits, disease treatment methods of gastric cancer patients and the detection methods for *H.pylori* infection status might influence the association. In addition, it has been widely known that the *H.pylori* strains between the Asian and non-Asian populations were different, especially for the virulence protein cagA of *H.pylori* [62]. cagA of the Asian populations infected *H.pylori* strains leads to more severe immune responses and epithelial cytoskeletal changes in gastric epithelial cells [63]. These might contribute to the different associations between *H.pylori* infection status and outcomes of gastric cancer patients in the Asian and non-Asian population. In the stratification analysis of the *H.pylori* detection methods, a significant association was noticed for those studies conducted with the serologic analysis method or with two more detection methods but not for those studies only single method including histological or PCR examination. It has been reported that the colonization might be not suitable for *H.pylori* in the gastric cancer tissues as the pH might be raised due to the alkalization of the cells in the gastric cancer development [19]. The histological and PCR examination was usually conducted in the gastric cancer tissues instead of the other sites for *H.pylori* colonization, which might lead to false negative results for *H.pylori* infection tests. Therefore, two or more detection methods would be necessary to accurately determine the *H.pylori* status for gastric cancer patients in order to predict the outcomes of gastric cancer patients according to the *H.pylori* status. A negative but non-statistically significant association between the *H.pylori* infection status at diagnosis and the DFS for gastric cancer patients was noticed in the meta-analysis of 12 eligibility studies, suggesting more studies with larger sample size are warranted to elucidate the influences of *H.pylori* on the disease progression of the gastric cancer patients.

We acknowledged there were several limitations for the current meta-analysis should to be stated. First, there was significant heterogeneity between the studies, which may be caused by study-specific characteristics. Stratified analyses only partially reduced the heterogeneity between the studies as the subgroup studies also found significant heterogeneity between the studies. We also applied the Baujat plot to identify those studies that largely contribute to the heterogeneity between the studies, and found the association between *H.pylori* infection and OS was still significant after excluding those studies, suggesting that the results were robust. Secondly, the strains of *H. pylori* should be determined for infected gastric cancer patients, as the bacteria strains between the non-Asian populations and the Asian populations are different, and the host immune responses for *H. pylori* infection between the strains depends on the cag pathogenicity island of the bacteria [64]. Strain-specific effects of *H. pylori* on gastric cancer prognosis should be determined. At last, the pooled estimates were varied according to the *H.pylori* detection methods. Studies performed with the serologic analysis could not exclude the possibility that a previous *H.pylori* infection history but not the positive status at diagnosis was associated with the OS of gastric cancer patients. Studies with combined sensitive *H.pylori* detection methods are warranted to further address the related questions in future.

In conclusion, the current study reported that gastric cancer patients with *H. pylori* infections have better outcomes in relative to those without infection at diagnosis. Unbiased studies with larger sample sizes are warranted to validate the conclusions, and the underlying mechanisms need to be explored with more studies.

## MATERIALS AND METHODS

### Literature search

Eligible studies were identified by searching the PubMed and MEDLINE databases published up to March 1^st^, 2017 following the Preferred Reporting Items for Systematic Reviews and Meta-Analyses (PRISMA) guidelines. The following terms (1) “gastric” OR “stomach” OR “cardia”; (2) “cancer” OR “carcinoma” OR “neoplasia” OR “adenocarcinoma”; (3) “*Helicobacter pylori*” OR “*H. pylori”*; (4) “outcome” OR “prognosis” OR “survival” were used to identify studies that evaluated the association between *H. pylori* infection status and outcomes for gastric cancer patients. In addition, we also checked the references of the identified eligibility studies to search for potential missing studies. Only those studies published in English were included in the current study. The EndNote software (version X7.4, Thomson Reuters) was applied to detect the repeated reports in the two databases and retrieve the title and abstract of the reports.

### Eligibility study criteria

Standardized inclusion criteria were applied to the retrieved reports. First, the study should be of case-cohort design and evaluated the *H.pylori* status at the time of diagnosis (positive or negative); Second, the outcomes for the gastric cancer patients including the OS, cancer-specific mortality, relapse-free survival or DFS should be compared between the groups of *H.pylori* status (positive *vs*. negative); Third, each study should classify the *H. pylori* status for the gastric cancer patients at diagnosis and provide the correlating estimated HR and it corresponding 95% CI for the outcomes under the Cox proportional hazard model (univariate or multiple variate analyses) or provided a Kaplan-Meier plot that could be used to calculate the HR and its 95% CI estimate for patients. We excluded those studies if they: 1) with overlapping participants with other studies but of relative smaller sample size; 2) did not provide sufficient information to calculate the risk estimate for H.pylori positive *vs*. negative patients; 3) were not published as full reports (e.g. conference abstracts).

### Data extraction

The following data were extracted from each study: last name of the first author, year of publication, study country, sample size, the disease stage of patients, the median and the range of following-up time, number of patients with positive *H. pylori* status, methods for *H. pylori* detection, the estimated HRs and their corresponding 95% CIs for the OS, cancer specific mortality, DFS or relapse-free survival of the gastric cancer patients, and whether the risk estimate was adjusted for covariates.

### Eligibility study quality assessment

For the eligible cohort studies, the quality assessment was performed with the modified Newcastle-Ottawa quality assessment scale [65]. A total of ten points was designated according to three broad perspectives, including the selection of the study groups (five points), the comparability of the groups (two points), and the ascertainment of either the exposure or outcome of interests for cohort studies respectively (three points). In the study selection category, five points were awarded if the study were performed with clearly diagnosed as gastric cancer (one point), compared the outcomes for *H.pylori* positive *vs*. negative status (one point), *H.pylori* status detected at the diagnosis (one point), and provided the diagnosis method (0 point for not reported, 1 point for single detection method and 2 points for studies with two or more detection methods). In the comparability category, a maximum 2 points were awarded if the risk estimate has been adjusted for the covariates of the patients. 0 point was awarded for those studies with the risk estimate was calculated based on the provided information, while 1 point was awarded for study provided the univariate risk estimate. For the outcome category, three points were awarded if the study concerned the OS or the DFS of the patients, with median following-up time more than 36 months and with sufficient following-up rate (Supplementary Table 1). Studies with total score > 7 were recognized as with higher quality. Two authors (XF and KL) independently assessed the study quality and disagreements were resolved through discussing with the third author (PC).

### Statistical methods for meta-analysis studies

The estimated HRs with their corresponding 95% CIs for the *H. pylori*-positive in contrast to *H. pylori*-negative status were used to calculate the pooled hazard ratio estimates for OS or DFS of the gastric cancer patients. For those studies only provided a Kaplan-Meier plot of the gastric cancer patients outcomes that stratified by the *H. pylori* status, the survival curve was reconstructed with Engauge Digitizer software (version 4.1, GitHub, Inc.), and the plot was used to calculate the estimated HR and its 95% CI, according to the method proposed by Tierney et al. [66]. For the other studies that provided the HR point estimate, the group size and the observed events or the log-rank statistic results for Kaplan-Meier plot, the HR estimate and its 95% CI were calculated following the methods provided by Tierney et al. [66]. Estimates for relapse-free survival and cancer-specific survival were assumed to be the DFS and the OS in the current study, respectively.

In the meta-analysis, to establish appropriate weighting for each study, the SE for each logarithm HR (logHR) was calculated and recognized as the estimated variance for the logHR. The generic inverse variance approach was applied for weighting for each individual study. The DerSimonian and Laird random-effects model, which considers the variability both within and between studies, was applied to calculate the pooled estimate and its 95% confidential interval (95% CI) [67]. Statistical heterogeneity between the studies was quantified with the Cochrane Q-test, together with I^2^ statistics (significance set at *I*^2^ > 25%). We also applied a graphical method proposed by Baujat et al. to identify those studies that contribute mostly to the heterogeneity between the studies [68]. Meta-regression method in together with the stratification analysis was applied to determine whether the pooled estimates could be influenced by the study level characteristics. Publication bias was represented as funnel plots and further assessed by the Egger’s linear test and Begg’s rank correlation test [69]. Sensitivity studies were performed by excluding individual studies and calculated the pooled estimates for the left studies repeatedly to identify the individual studies that significantly affected the overall estimates. The cumulative meta-analyses were also performed to chronologically determine the stabilization of the pooled estimates under the random-effects model.

For all statistical analysis, the P-value < 0.05 were considered as statistically significant for two-sided test. R software (version 3.1.1, www.r-project.org) and Revman (version 5.0) were used for all statistical analyses. The systematic review was performed and reported following the MOOSE guidelines (Supplementary Table 2).

## SUPPLEMENTARY MATERIALS FIGURES AND TABLES




