# Supplementary material for: Positive Helicobacter pylori status is associated with better overall survival for gastric cancer patients: evidence from case-cohort studies
Source: Oncotarget. 2017 Jun 28;8(45):79604–17. doi: 10.18632/oncotarget.18758 (PMC5668073; doi:10.18632/oncotarget.18758)
Supplement: Supplementary file 1 [file oncotarget-08-79604-s001.pdf]

Positive *Helicobacter pylori* status is associated with better overall survival for gastric cancer patients: evidence from case-cohort studies

SUPPLEMENTARY MATERIALS

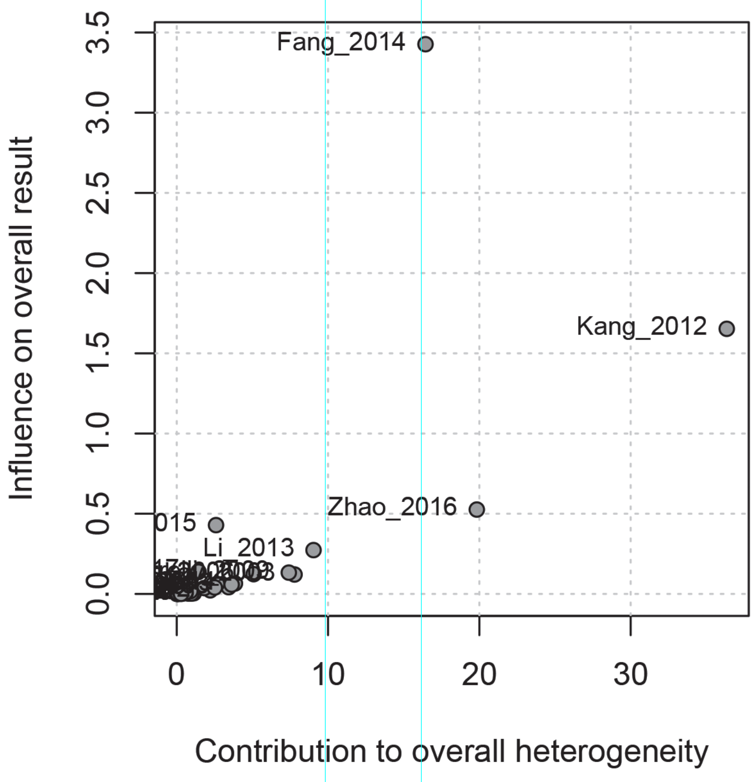

Supplementary Figure 1: The Baujat plot for the studies assessed the association between the H.pylori infection status at diagnosis and overall survival of gastric cancer patients. The x-axis indicated the contribution of each study to the overall heterogeneity between the studies, while the y-axis suggested the influence of each study on the overall result.

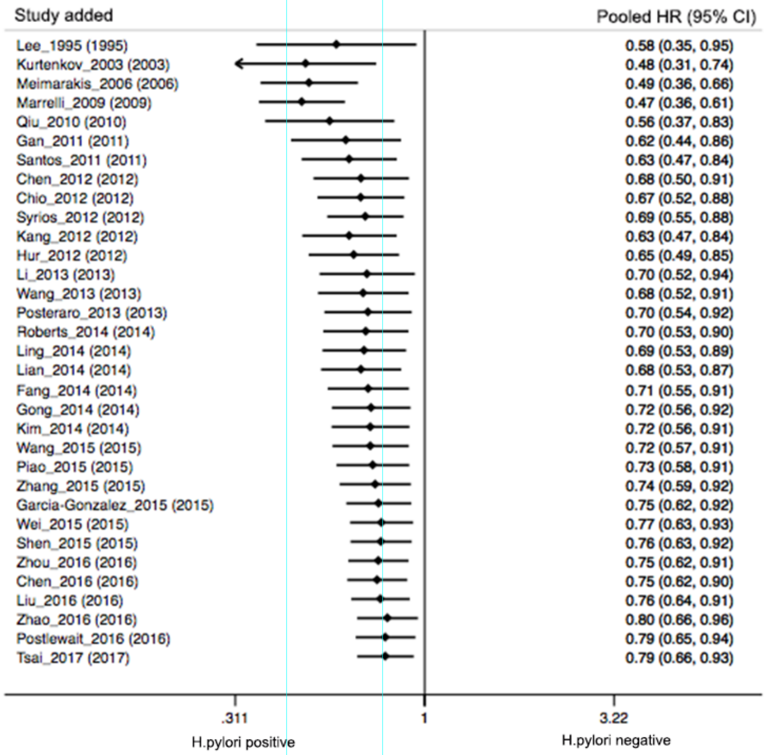

Supplementary Figure 2: The cumulative meta-analysis of the studies determined the association between the H.pylori infection at diagnosis (positive vs. negative) and overall survival of gastric cancer patients.

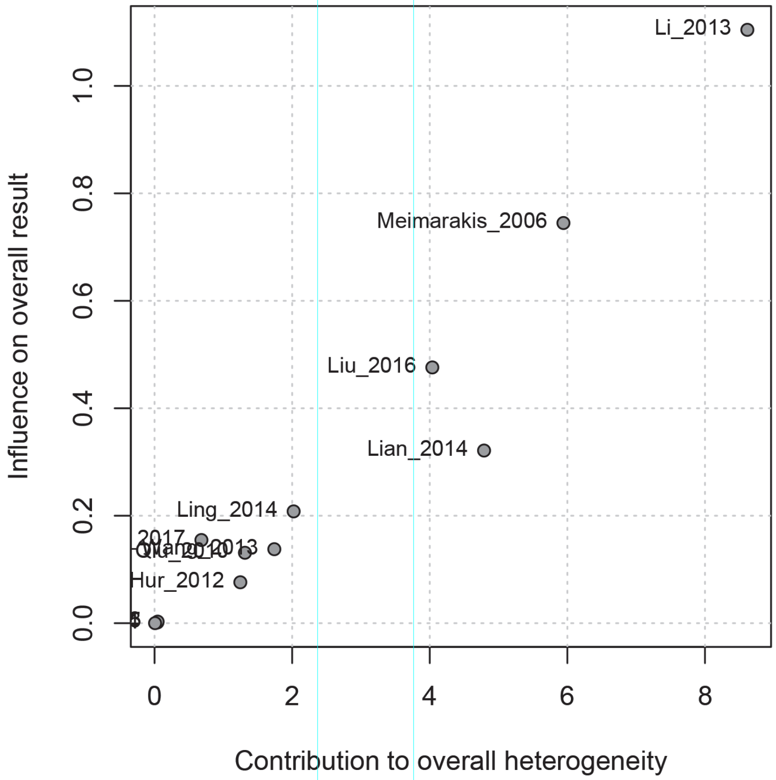

Supplementary Figure 3: The Baujat plot for the studies that had assessed the association between the H.pylori infection status at diagnosis and disease-free survival for gastric cancer patients. The x-axis indicated the contribution of each study to the overall heterogeneity between the studies, while the y-axis suggested the influence of each study on the overall result.

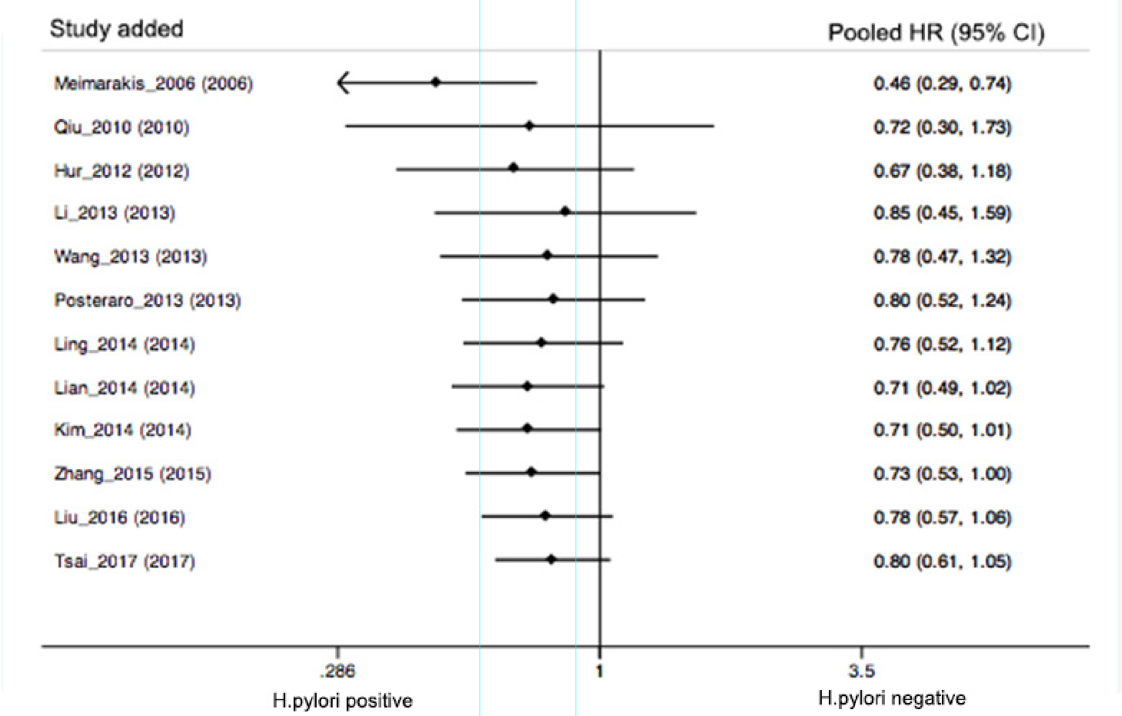

Supplementary Figure 4: The cumulative meta-analysis of the studies determined the association between the H.pylori infection at diagnosis (positive vs. negative) and disease-free survival of gastric cancer patients.

For Supplementary Tables see in Supplementary Tables
Supplementary Table 1: The quality assessment of the 33 included studies for the meta-analysis studies.
Supplementary Table 2: The MOOSE statement for the current meta-analysis study.
