# Supplementary material for: Positive Helicobacter pylori status is associated with better overall survival for gastric cancer patients: evidence from case-cohort studies
Source: Oncotarget. 2017 Jun 28;8(45):79604–17. doi: 10.18632/oncotarget.18758 (PMC5668073; doi:10.18632/oncotarget.18758)
Supplement: Supplementary file 2 [file oncotarget-08-79604-s002.docx]

**Supplementary Table 1.** The quality assessment of the 33 included studies for the meta-analysis studies.

| Source | Representativeness of the exposed cohort | Selection of the unexposed cohort | Ascertainment of exposure* | H.pylori detection at diagnosis | Control for important factor or additional factor^#^ | Outcome assessment | Follow-up long enough for outcomes to occur^&^ | Adequacy of follow-up of cohorts | Total quality scores |
| --- | --- | --- | --- | --- | --- | --- | --- | --- | --- |
| Lee *et al*, 1995 | ☆ | ☆ | ☆ | ☆ | — | ☆ | ☆ | ☆ | 7/10 |
| Kurtenkov *et al*, 2003 | ☆ | ☆ | ☆ | ☆ | — | ☆ | — | ☆ | 6/10 |
| Meimarakis *et al*, 2006 | ☆ | ☆ | ☆☆ | ☆ | ☆☆ | ☆ | ☆ | ☆ | 10/10 |
| Marrelli *et al*, 2009 | ☆ | ☆ | ☆☆ | ☆ | ☆☆ | ☆ | ☆ | ☆ | 10/10 |
| Qiu *et al*, 2010 | ☆ | ☆ | ☆ | ☆ | — | ☆ | — | ☆ | 6/10 |
| Gan *et al*, 2011 | ☆ | ☆ | ☆ | ☆ | ☆☆ | ☆ | ☆ | ☆ | 9/10 |
| Santos *et al*, 2011 | ☆ | ☆ | ☆ | ☆ | — | ☆ | ☆ | ☆ | 7/10 |
| Syrios *et al*, 2012 | ☆ | ☆ | ☆ | ☆ | — | ☆ | — | ☆ | 6/10 |
| Kang *et al*, 2012 | ☆ | ☆ | ☆ | ☆ | ☆☆ | ☆ | ☆ | ☆ | 9/10 |
| Chen *et al*, 2012 | ☆ | ☆ | ☆ | ☆ | ☆ | ☆ | ☆ | ☆ | 8/10 |
| Chio *et al*, 2012 | ☆ | ☆ | ☆ | ☆ | — | ☆ | — | ☆ | 6/10 |
| Hur *et al*, 2012 | ☆ | ☆ | ☆☆ | ☆ | — | ☆ | — | ☆ | 7/10 |
| Wang *et al*, 2013 | ☆ | ☆ | ☆ | ☆ | ☆☆ | ☆ | ☆ | ☆ | 9/10 |
| Supplementary Table 1. Continued. | | | | | | | | | |
| Source | Representativeness of the exposed cohort | Selection of the unexposed cohort | Ascertainment of exposure* | H.pylori detection at diagnosis | Control for important factor or additional factor^#^ | Outcome assessment | Follow-up long enough for outcomes to occur^&^ | Adequacy of follow-up of cohorts | Total quality scores |
| Li *et al*, 2013 | ☆ | ☆ | ☆ | ☆ | ☆☆ | ☆ | — | ☆ | 8/10 |
| Posteraro *et al*, 2014 | ☆ | ☆ | ☆ | ☆ | ☆ | ☆ | ☆ | ☆ | 8/10 |
| Gong, 2014 | ☆ | ☆ | ☆ | ☆ | ☆ | ☆ | ☆ | ☆ | 8/10 |
| Fang *et al*, 2014 | ☆ | ☆ | — | ☆ | ☆☆ | ☆ | — | ☆ | 7/10 |
| Roberts *et al*, 2014 | ☆ | ☆ | ☆ | ☆ | ☆ | ☆ | — | ☆ | 7/10 |
| Ling *et al*, 2014 | ☆ | ☆ | — | ☆ | ☆☆ | ☆ | — | ☆ | 7/10 |
| Lian *et al*,  2014 | ☆ | ☆ | ☆ | ☆ | ☆☆ | ☆ | — | ☆ | 8/10 |
| Kim *et al*,  2014 | ☆ | ☆ | ☆☆ | ☆ | ☆☆ | ☆ | — | ☆ | 9/10 |
| Shen *et al*,  2014 | ☆ | ☆ | — | ☆ | ☆ | ☆ | — | ☆ | 6/10 |
| Garcia-Gonzalez et al, 2015 | ☆ | ☆ | ☆☆ | ☆ | ☆ | ☆ | — | ☆ | 8/10 |
| Piao et al, 2015 | ☆ | ☆ | ☆ | ☆ | ☆ | ☆ | — | ☆ | 7/10 |
| Wei et al, 2015 | ☆ | ☆ | — | ☆ | ☆ | ☆ | — | ☆ | 6/10 |
| Wang et al, 2015 | ☆ | ☆ | ☆☆ | ☆ | ☆ | ☆ | — | ☆ | 8/10 |
|  |  |  |  |  |  |  |  |  |  |
| Supplementary Table 1. Continued. | | | | | | | | | |
| Source | Representativeness of the exposed cohort | Selection of the unexposed cohort | Ascertainment of exposure* | H.pylori detection at diagnosis | Control for important factor or additional factor^#^ | Outcome assessment | Follow-up long enough for outcomes to occur^&^ | Adequacy of follow-up of cohorts | Total quality scores |
| Zhang et al, 2015 | ☆ | ☆ | ☆ | ☆ | ☆☆ | ☆ | — | ☆ | 8/10 |
| Zhao et al, 2016 | ☆ | ☆ | — | ☆ | ☆ | ☆ | — | ☆ | 6/10 |
| Zhou et al, 2016 | ☆ | ☆ | ☆ | ☆ | ☆ | ☆ | ☆ | ☆ | 8/10 |
| Chen et al, 2016 | ☆ | ☆ | — | ☆ | ☆ | ☆ | — | ☆ | 6/10 |
| Postlewait, 2016 | ☆ | ☆ | — | ☆ | ☆☆ | ☆ | ☆ | ☆ | 8/10 |
| Liu et al, 2016 | ☆ | ☆ | — | ☆ | ☆☆ | ☆ | — | ☆ | 7/10 |
| Tsai et al, 2017 | ☆ | ☆☆ | ☆ | ☆ | ☆☆ | ☆ | — | ☆ | 9/10 |
|  | | | | | | | | | |

^*^Two points were awarded if the study performed with two or more H.pylori detection methods, one point was awarded if one detection method was applied, and zero point for those study did not provide the detection method.

^#^Two points were awarded if the study important confounders such as disease stage, sex, age, grade etc., one star was awarded if the study only provided the univariate analysis results, and zero for those study with the estimate was recalculated based on the provided information.

^&^Cohort study with a follow-up time ≥ 3 years or long enough for outcomes to occur was assigned one star.

**Supplementary Table 2.** The MOOSE statement for the current meta-analysis study

| **Criteria** | | **Brief description of how the criteria were handled in the meta-analysis** |
| --- | --- | --- |
| **Reporting of background should include** | |  |
| √ | Problem definition | *Helicobacter pylori* (*H. pylori*) infection is a risk factor for gastric cancer; however, the associations between the infection status and prognosis for gastric cancer patients are controversial. In the current study, we determined such associations in case-cohorts by meta-analysis methods. |
| √ | Hypothesis statement | Positive *H. pylori* status may be associated with the better outcomes for the gastric cancer patients. |
| √ | Description of study outcomes | Hazard ratios (HRs) for overall survival, and disease-free survival for the gastric cancer patients. |
| √ | Type of exposure or intervention used | The *H. pylori* status of the gastric cancer patients. |
| √ | Type of study designs used | In this study, we included case-cohort studies that have evaluated the association of the *H. pylori* status at diagnosis and outcomes of the gastric cancer patients. |
| √ | Study population | We placed no restriction. |
| **Reporting of search strategy should include** | |  |
| √ | Qualifications of searchers | The credentials of the two investigators X.F and K.L are indicated in the author list. |
| √ | Search strategy, including time period included in the synthesis and keywords | PubMed from 1990 – March, 2017  MEDLINE from 1990 –March, 2017  The terms used as indicated in the Methods part. |
| √ | Databases and registries searched | PubMed and MEDLINE. |
| √ | Search software used, name and version, including special features | No special software was used. However, EndNote was used to check the retrieved citations and eliminate duplications |
| √ | Use of hand searching | References were also checked to identify any missing studies. Details of the study were thoroughly examined in order to exclude potentially overlapping data. |
| √ | List of citations located and those excluded, including justifications | The detailed literature research process was shown in Figure 1. |
| √ | Method of addressing articles published in languages other than English | Paper published in languages other than English was not included in the current study. |
| √ | Method of handling abstracts and unpublished studies | Abstracts were thoroughly reviewed. |
| √ | Description of any contact with authors | None. |
| **Reporting of methods should include** | |  |
| √ | Description of relevance or appropriateness of studies assembled for assessing the hypothesis to be tested | Detailed inclusion and exclusion criteria were described in the Figure 1. |
| √ | Rationale for the selection and coding of data | Data extracted from each of the studies were relevant to the study characteristics including last name of the first author, year of publication, study country, study design, sample size, the median or mean follow-up time, number of patients with positive *H. pylori* status, methods for *H. pylori* detection, and the estimated HRs and corresponding 95% CIs for the overall survival or the disease-free survival for gastric cancer patients (positive vs. negative status of *H. pylori* infection). |
| √ | Assessment of confounding | The risk estimates that reflected the greatest degree of control for potential confounders were extracted from individual studies. Otherwise, the risk estimate was calculated based on the raw data provided by the authors. |
| √ | Assessment of study quality, including blinding of quality assessors; stratification or regression on possible predictors of study results | The Newcastle-Ottawa quality assessment scale was performed to assess the quality of the included study. The stratification studies were also performed according to potential factors that may affect the association between the *H. pylori* status and the outcomes of the gastric cancer. |
| √ | Assessment of heterogeneity | Heterogeneity of the studies was evaluated using the Cochrane’s Q test and I^2^ statistic. The Baujat plot was also applied to identify those studies largely contributed to the heterogeneity between the studies. |
| √ | Description of statistical methods in sufficient detail to be replicated | The meta-analysis methods were described in the Materials and Methods parts. |
| √ | Provision of appropriate tables and graphics | The Figure 1 showed the flow chart of the selection procedures. The Figure 2 to 5 showed the results of the meta-analysis. Table 1 provided detailed information for the included studies. Table 2 and 3 provided results from sensitivity tests and subgroup analysis. |
| **Reporting of results should include** | |  |
| √ | Graph summarizing individual study estimates and overall estimate | Figure 2 and Figure 4. |
| √ | Table giving descriptive information for each study included | Table 1, Supplementary Table 1. |
| √ | Results of sensitivity testing | No significant publication bias was found for the included studies suggested by the Egger’s test. No single study significantly affects the results of the studies in the sensitivity studies. Table 2 and Table 3 provided the detailed meta-analysis results. |
| √ | Indication of statistical uncertainty of findings | 95% confidence intervals were presented for all summary estimates. The heterogeneity between the studies was evaluated and the publication bias was tested. |
| **Reporting of discussion should include** | |  |
| √ | Quantitative assessment of bias | Heterogeneity between the studies was evaluated and the publication bias was tested. |
| √ | Justification for exclusion | Papers didn’t evaluate the association for *H. pylori* status and outcomes for gastric cancer patients were excluded from the study. |
| √ | Assessment of quality of included studies | We conducted the sensitivity analyses to identify the individual study that may affect the overall pooled estimates and the heterogeneity between the studies was evaluated with the Q-test and the I^2^ statistic. The Newcastle-Ottawa quality assessment scale was performed to assess the quality of the included study. |
| **Reporting of conclusions should include** | |  |
| √ | Consideration of alternative explanations for observed results | H. pylori infection induced immune response, and H. pylori infection induced signal pathways, protein secreted by *H. pylori* (HP-NAP), all these together could explain the potentially protective effect of H. pylori infection on prognosis of gastric cancer patients. *H. pylori* Negative H. pylori status is usually associated with the advanced disease stage and the histopathological changes such as atrophy and intestinal metaplasia in gastric mucosa, which is no longer suitable for *H. pylori* survival in the microenvironment, which may be partially attribute to the association between *H. pylori* and outcomes for the gastric cancer patients. |
| √ | Generalization of the conclusions | In conclusion, the present meta-analysis shows that gastric cancer patients with *H. pylori* infections have a better prognosis relative to those without infections. More studies with larger sample sizes are warranted to validate the conclusions, and the underlying mechanisms need to be elucidated. |
| √ | Guidelines for future research | More well-designed studies with larger sample size, longer follow-up and more detailed patients information concerning previous *H. pylori* infection history are needed to validate whether positive *H. pylori* status are associated with better prognosis. |
| √ | Disclosure of funding source | The work was financially supported by grants from the Science and Technology Commission of Shanghai Municipality (14391901800), and the Shanghai Municipal Commission of Health and Family Planning (20164Y0250). |
